# Clinical Significance of CD147 in Children with Inflammatory Bowel Disease

**DOI:** 10.1155/2020/7647181

**Published:** 2020-09-15

**Authors:** Hongli Wang, Jun Ye, Ruitao Liu, Guanhua Chen, Junhong Zhao, Ling Huang, Fangying Yang, Musheng Li, Shunxian Zhang, Jingxie, Liya Xiong, Huan Chen, Yuxin Xu, Mingmin Su, Yuanwen Xie, Songyu Li, Fengfeng Zheng, Lanlan Geng, Wanfu Xu, Sitang Gong

**Affiliations:** ^1^The First Affiliated Hospital, Jinan University, Guangzhou, China; ^2^Department of Gastroenterology, Guangzhou Women and Children's Medical Center, Guangzhou Medical University, Guangzhou, China; ^3^Department of Anesthesiology, Zhuhai Maternal and Child Health Hospital, Zhuhai, China; ^4^Department of Preventive Medicine, School of Public Health, Fujian Medical University, Fuzhou, China; ^5^Department of Cancer Biology and Therapeutics, School of Pharmacy and Pharmaceutical Sciences, Cardiff University, Wales, CF103AT, UK; ^6^Department of Anorectal, Qionghai Hospital of Traditional Chinese Medicine, Qionghai, China; ^7^Department of Clinical Laboratory, Qionghai Hospital of Traditional Chinese Medicine, Qionghai, China; ^8^Department of infectious diseases, The Affiliated Hospital of Putian University, Putian, China

## Abstract

**Background:**

CD147/basigin (Bsg), a transmembrane glycoprotein, activates matrix metalloproteinases and promotes inflammation.

**Objective:**

The aim of this study is to explore the clinical significance of CD147 in the pathogenesis of inflammatory bowel disease (IBD).

**Results:**

In addition to monocytes, the clinical analysis showed that there is no significance obtained in leucocyte, neutrophil, eosinophil, basophil, and erythrocyte between IBD and controls. Immunohistochemistry analysis showed that CD147 was increased in intestinal tissue of patients with active IBD compared to that in the control group. What is more, CD147 is involved in intestinal barrier function and intestinal inflammation, which was attributed to the fact that it has an influence on MCT4 expression, a regulator of intestinal barrier function and intestinal inflammation, in HT-29 and CaCO2 cells. Most importantly, serum level of CD147 content is higher in active IBD than that in inactive IBD or healthy control, which could be a biomarker of IBD.

**Conclusion:**

The data suggested that increased CD147 level could be a biomarker of IBD in children.

## 1. Introduction

Multiple studies have showed that inflammatory bowel disease (IBD) is a disorder of dysregulation by inflammation accompanied by impaired intestinal barrier function, which is an vital event in treatment of IBD [1, 2]. The local inflammatory reaction is involved in the recruitment of various inflammatory cells and secretion of cytokines, including matrix metalloproteinases (MMPs), IL-1*β*, and IL-6, which have a critical role in regulation of intestinal barrier function, thereby resulting in aggravating development of IBD. Thus, deciphering the key molecules that regulate intestinal barrier function and inflammatory reaction is urgently needed to improve therapeutic outcomes.

CD147, also named EMMPRIN, is an abundant 269aa type 1 integral glycosylated and multifunctional membrane protein with different cellular functions on differing cell subpopulations. Multiple studies have showed that CD147 is expressed within a wide range of tissues, including endothelium and epithelium. CD147 is thought to act via MAPK p38, ERK-1/2, PI3K, and NF-*κ*B signaling pathways [3–5]. The biological function of CD147 in tumor may be through the regulation of matrix metalloproteinase (MMPs) and monocarboxylate transporter (MCT), which has been well documented [6]. MMPs, especially highly elevated MMP-9 in patients with IBD, increase in intestinal epithelial tight permeability through the P38 kinase signaling pathway-mediated myosin light chain kinase (MLCK) gene [7] and was responsible for the inflammatory response and tissue damage in mouse models of colitis [8]. Moreover, the MMP-9 level is markedly elevated in intestinal tissue, serum, and stool of patients with IBD and closely correlates with the disease activity and degree of inflammation [9, 10], while plasma matrix metalloproteinase-1 (MMP-1) and tissue inhibitor of metalloproteinase-1 (TIMP) were demonstrated to increase in the peripheral blood sample of patients with IBD, suggesting a positive correlation with endoscopic mucosal injury, disease activity, clinical activity, and CRP [11].

In our pervious study, MCT4, a chaperone of CD147, has been reported to increase in the intestinal mucosa in IBD [12], and alterations of MCT4 expression are sufficient to induce a switch between CBP-NF-*κ*B and CBP-CREB complex, leading to different biological functions in inflammatory bowel disease, and treatment of experimental colitis with MCT4 inhibitor *α*-cyano-4-hydroxycinnamate (CHC) ameliorated mucosal intestinal barrier function, which was due to attenuation of proinflammation factor expression and enhancement of ZO-1 expression [12, 13]. Despite the strong research concerning the function of CD147 in tumor, data is lacking in inflammatory bowel disease, especially in children. In this study, we aimed to seek evidence to elucidate the possible relationship between CD147 expression and IBD development as well as prognosis.

## 2. Materials and Methods

### 2.1. Patients and Biopsies

Based on the Declaration of Helsinki as reflected in a prior approval by the institution's human research committee, this study was conducted in a cohort of children patients with IBD in Guangzhou Women and Children's Medical Center approved by the Medical Ethical Review Board, named Scientific Research Committee of Guangzhou Women and Children's Medical Center (the clinical trial registration number is ChiCTR1800015160).

A total of 77 cases with IBD and 19 cases of control subjects were included in this study; the detailed information was supplied in Supplementary Materials. The intestinal tissue was drawn from each patient by electronic colonoscopy after we got the informed consent from the patients diagnosed with IBD. The clinical activity was determined as in Chen et al.'s study [14]. Written informed consent was given by the caregiver of the child for his clinical records used, which are not publicly available since the database is currently not anonymous and contains all patient's name; however, it could be available upon request.

### 2.2. Blood Gas Analyses

Blood samples were collected from patients for analysis in clinical laboratory.

### 2.3. Immunohistochemistry

We followed the methods of Zhang et al.'s study. Colonic tissue from patients was routinely formalin-fixed and paraffin-embedded and then cut into slices of 4 mm for immunohistochemical staining. To test the CD147 expression in intestinal epithelium of IBD patients, the sections were processed with the following steps. Slices were placed in a 55°C oven for 2 hours and then deparaffinized in xylene and in gradient alcohol solutions. 1× citrate buffer (heated to boiling) was used for antigen retrieval. When cooled to room temperature, hydrogen peroxide (3%) was used to inactivate endogenous peroxide activity, blocked in goat serum at room temperature for 40 min, followed by incubation with anti-CD147 (Abagent, AP73654, 1 : 1000 for WB and 1 : 200 for IHC) antibody overnight at 4°C. After being colorized with diaminobenzidine (DAB Kit; ZSGB-BIO Co) and counterstained with hematoxylin, sections were dehydrated in gradient alcohol solutions and in xylene. Section mounting of the coverslips was photographed by microscopy.

### 2.4. Enzyme-Linked Immunosorbent Assay

As described in Zhang et al.'s study, briefly, blood specimens were adequately centrifuged for extracting serum specimens. Serum specimens were stored at -80°C until analysis. Serum level of CD147 was detected using a commercial human EMMPRIN/CD147 Quantikine ELISA Kit (catalogue no. DEMP00; R&D Systems, Minneapolis, MN, USA) according to a manufacturer's instructions [17].

### 2.5. Cell Culture and Transfection

Intestinal epithelial cells HT-29 and CaCO2 were cultured in DMEM supplemented with 10% fetal bovine serum and maintained in a humidified incubator at 37°C and 5% CO_2_. For transfection, plasmids, purchased from GenScript company, or siRNA was transfected into cells with HilyMax (H357, Dojindo, Kamimashikigun, Kumamoto, Japan) and Lipofectamine 3000 (Invitrogen, Carlsbad, CA, USA), respectively, following the manufacturer's instructions.

### 2.6. Western Blotting

As described in our previous study [15], protein concentrations were determined using Pierce BCA Protein Assay Kit (Thermo Fisher Scientifics) and prepared in 2x SDS sample buffer and subjected from SDS-PAGE and transferred to a 0.22 *μ*m nitrocellulose transfer membrane. The membrane was blocked with 5% (*w*/*v*) milk in PBS/0.05% (*v*/*v*) Tween-20 and incubated with the indicated antibody overnight at 4°C followed by incubation with a horseradish peroxidase secondary antibody (Jackson ImmunoResearch) for 1 h at room temperature. Proteins were detected using an enhanced chemiluminescence (Perkin Elmer).

### 2.7. Real-Time PCR

As described in our studies [13, 15], total RNA was extracted using Trizol (Life Technologies) and converted to cDNA using the All-in-One™ First-Strand cDNA Synthesis Kit (Genecopoeia™, FulenGen) and amplified by PCR using the All-in-One™ qPCR Mix (Genecopoeia™, FulenGen) according to the manufacturer's instructions. The primer sequences are as follows: for MCT4: GTCATCTCTCTGCCCCACAT (sense), AGCACGGTCAATGAGAACAA (antisense); CD147: GGCTGTGAAGTCGTCAGAACAC (sense) and ACCTGCTCTCGGAGCCGTTCA (antisense); and GAPDH: GGTATGACAACGAATTTGGC (sense), reverse: GAGCACAGGGTACTTTATTG (antisense).

### 2.8. Statistical Analysis

All statistical analyses were performed using SPSS 22.0 (SPSS, Inc., Chicago, III). Data were expressed at the mean with standard deviation (SD). *t*-test was used to analyze the difference of the serum CD147 level. All statistical analyses utilized a 0.05 level of significance.

## 3. Results

### 3.1. Patient Characteristics

Clinical and biochemical analyses of the study cohort are listed in Supplementary Materials. As shown in Table 1, the subjects in this study consisted of 63 (65.6%) boys and 33 (34.4%) girls with an age at diagnosis of 1 to 18 years (median, 9 years) in Guangzhou Women and Children's Medical Center. With regard to IBD grade, patients were classified as active and in the remission stage, 80.2% and 19.8%, respectively. In addition, the median age of remission subjects (*n* = 19) and IBD subjects (*n* = 77) was 5.9 years and 9.6 years, respectively. 95% patients (73 cases) were Crohn's disease (CD) patients, while 5% patients (4 cases) were ulcerative colitis (UC) patients, respectively.

**Figure 1:**
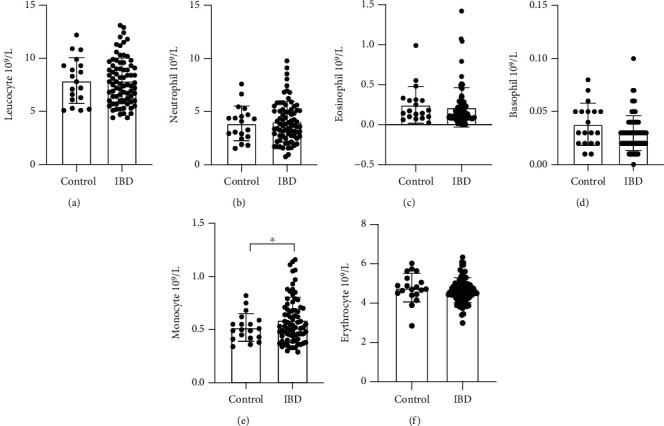
Clinical characteristic of patients. Statistical analysis of indicated index in patients with control (inactive stage) and active IBD.

As shown in Figures 1(a)–1(e), the results from blood gas analysis showed that there was no significant difference in leucocyte, 7.90 ± 2.16 vs. 7.869 ± 2.15∗10^9^; neutrophil 3.89 ± 1.63 vs. 4.06 ± 1.90∗10^9^; leukomonocyte, 3.19 ± 1.32 vs. 2.96 ± 1.29∗10^9^; eosinophil, 0.25 ± 0.22 vs. 0.22 ± 0.24∗10^9^; and basophilic granulocyte, 0.08 ± 0.02 vs. 0.029 ± 0.02∗10^9^ between control (*n* = 19) and IBD (*n* = 77), while there was a higher mononuclear leucocyte in IBD compared to that in control (0.61 ± 0.25 vs. 0.52 ± 0.13∗10^9^, *p* = 0.011) (Figure 1(f)).

### 3.2. Epithelial CD147 Expression Is Increased in Patients with IBD

**Figure 2:**
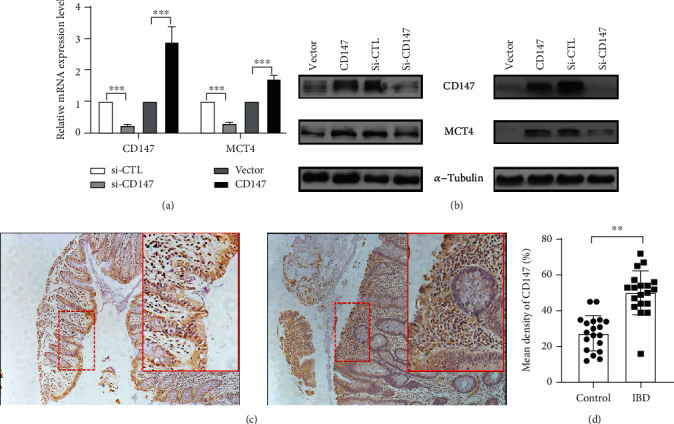
Evaluated CD147 is increased in intestinal mucosa. (a, b) Western blotting and real-time PCR were performed to detect the effect of CD147 on MCT4 expression in IECs. Immunohistochemical analysis was employed to examine CD147 expression in the indicated group (c), and relative density of CD147 was analyzed by *t*-test analysis (d).

Our previous studies showed that MCT4, an evaluated molecular and potential diagnostic marker in the intestinal mucosa of patients with inflammatory bowel disease, destroys intestinal barrier function by inhibition of cAMP-response element binding protein- (CREB-) mediated ZO-1 and activation of NF-*κ*B-induced IL-6 expression [12, 13], indicating the critical role of the MCT4-mediated pathway in the development of IBD. Interestingly, further work showed that CD147 drastically increased MCT4 mRNA level in CaCO2 of IECs; in line with this, the results showed that overexpression of CD147 enhanced MCT4 expression, while inhibition of CD147 significantly reduced MCT4 expression in HT-29 and CaCO2 cells (Figures 2(a) and 2(b)), implying involvement of CD147 in IBD in a MCT4-dependent way, which is consistent with the results that revealed that silencing either MCT1 or MCT4 in ARPE-19 cells resulted in decreased expression of their accessory subunit, CD147 [16, 17]. In addition, immunohistochemistry analysis was performed using a CD147 antibody to characterize the CD147 expression on the cellular level. The quantification exhibited that CD147 in intestinal epithelial cells was significantly higher in patients with IBD specimens (*n* = 77) relative to that in controls (*n* = 19, *p* < 0.01); what is more, we also found that enrichment of CD147 is majorly expressed in intestinal inflammatory cells, suggesting involvement of CD147 in intestinal inflammatory reaction (Figures 2(c) and 2(d)). Taken together, these results suggested a causal link between CD147 enhancement and IBD pathogenesis.

### 3.3. Serum CD147 Level Is Increased in IBD

**Figure 3:**
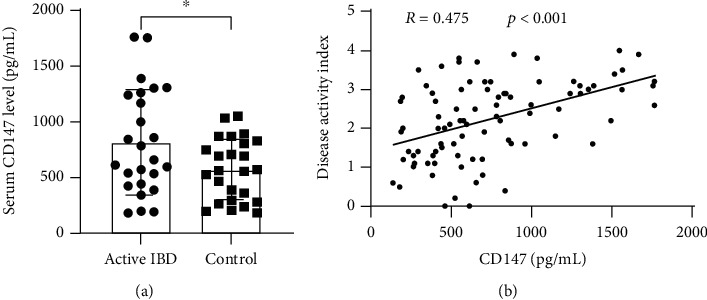
Serum CD147 level was measured in patients with IBD. (a) The blood was collected from patients in clinic, and ELISA for CD147 was measured to analyze its clinical significance. (B) The relationship was performed between DAI and serum CD147 level.

In addition to the transmembrane form, evidences have indicated that the presence of another form of CD147 has been detected in human serum and plasma where it is present as a soluble form (sCD147) derived from the proteolytic cleavage of the transmembrane receptor or from cell secretion [18, 19]. Next, we sought to measure the CD147 level in serum according to intestinal inflammation degree. As displayed in Figure 3(a), patients with active stage of IBD showed a higher serum CD147 level than those with inactive stage of IBD (*p* < 0.05), implying that the content of CD147 level is positively close with mucosal inflammation. What is more, further analysis showed the positive relationship between disease active index and the CD147 level (Figure 3(b)). All these findings suggested that CD147 could be a serum biomarker of IBD.

## 4. Discussion

A large number of studies have demonstrated that CD147 participated in the tumor progression, including proliferation [20], invasion and migration [21], and recurrence and prognosis [22], suggesting CD147 has a critical role in tumor progression. However, up to now, there are no available reports about the role of CD147 in inflammatory bowel disease. In this study, to our knowledge, we are the first to investigate the expression and clinical prognostic significance of CD147 in IBD. The expression of CD147 is increased in the intestinal mucosa of patients with IBD, and serum CD147, associated with DAI, is higher in inflamed patients than in noninflamed patients, which suggested that CD147 could be a serum biomarker in the IBD progress. However, in adult patients with CD (over 18 years) that had a confirmed diagnosis of CD based on clinical, endoscopic, and histologic data, the function of CD147 was used to identify patients in remission, based on endoscopic analysis, and monitor CD activity based on serum levels of proteins [23].

CD147 is an evolutionarily conserved member of the adhesion molecule family and widely expressed in human cells and plays an important role in many normal physiological functions [24]. It has been reported that MMPs, downstream effector of CD147 [25–27], were highly expressed in the intestinal mucosa of patients with IBD, and there is a significant correlation that has been established in UC patients, in particular between the increased expression of metalloproteinases and the examined histopathological markers which determine disease progression [7, 28–33]. For instance, CD147 could promote breast cancer cell metastasis and invasion by contribution of MMP2, MMP9, and VEGF expression [34], and CD147-mediated chemotaxis of CD4^+^CD161^+^ T cells may contribute to local inflammation in rheumatoid arthritis [35]. These results implied that CD147 involved in the development of IBD; however, the function of CD147 remained to be fully elucidated.

In our study, we found that CD147 is significantly increased in the intestinal mucosa of IBD analyzed by IHC, which possibly stemmed from the highly expressed MCT4, a regulator of such as VEGF, CD147, MMP2, and MMP9 [36], identified in our previous studies [12, 13]. In addition, CD147 remodeled microenvironment by regulating MMP2, collagen, hyaluronan, and MMP9 expression [37], which may promote intestinal fibroblast activation, leading to intestinal fibrosis. Further analysis showed that CD147 expression is positively correlated with intestinal inflammation, which is possibly attributed to proinflammatory factor, such as IL-18, which induces the MMP inducer EMMPRIN via JNK-dependent Sp1 activation [38]. In addition, CD147 promotes CXCL1 expression, which is a critical role in promoting innate inflammatory reaction. Of interest, several MMPs, highly expressed in the intestinal mucosa of IBD [7, 28], are regulated by CD147 a transmembrane glycoprotein receptor also found in a soluble plasmatic form [18]. As expected, serum CD147 is significantly increased in inflamed patients compared with that in inactive. These finding suggested the involvement of CD147 in inflammatory bowel disease. However, further work is required to elucidate the function of CD147 in the development of IBD, such as the influence of CD147 on the polaritons of macrophages into M1 or M2 macrophages or the intestinal barrier integrity. Targeting CD147 or blockade of CD147 signaling may be a novel therapeutic strategy for the treatment and prevention of IBD.

In summary, CD147 was significantly increased in the intestinal mucosa in patients with IBD and DSS-induced colitis, which is closely associated with intestinal inflammation. Most importantly, the serum CD147 level is higher in patients with active IBD compared to that in inactive IBD, implying a potential role of CD147 which acts as a biomarker of IBD.

## Figures and Tables

**Table 1:** The characteristic of patients with inflammatory bowel disease.

Variables	Number of subject (%)
Total	96
Age	
<10 years	54 (56.2)
≥10 years	42 (43.8)
Gender	
Male	63 (65.6%)
Female	33 (34.4%)
Type of IBD	
Crohn's disease	92(95%)
Ulcerative colitis	4 (5%)
Stage	
Active stage	77 (68.5)
Inactive stage	19 (31.5)

## Data Availability

The data used to support the findings of this study are available from the corresponding author upon reasonable request, which is attributed to the unpublicized clinical materials.

## Abbreviations

CD: Crohn's disease
CREB: cAMP-response element binding protein
CHC: *α*-Cyano-4-hydroxycinnamate
DSS: Dextran sulfate sodium
ERK1/2: Extracellular signal-regulated kinases 1 and 2
IBD: Inflammatory bowel disease
IECs: Intestinal epithelial cells
IL: Interleukin
MCT4: Monocarboxylate transporter 4
MMPs: Matrix metalloproteinases
NF-*κ*B: Nuclear factor-*κ*B
p38: p38 mitogen-activated protein kinase
UC: Ulcerative colitis
ZO-1: Tight junction protein 1.

## Supplementary Materials

Supplementary Materials S1: the detailed information of 96 subjects was listed.
